# Conditional noise generative adversarial networks with Siamese neural network for longer time series forecasting

**DOI:** 10.1038/s41598-025-30874-w

**Published:** 2025-12-02

**Authors:** Haotian Mao, Xiao Feng

**Affiliations:** 1 China Telecom Corporation Limited Jiangsu Branch, Nanjing, 210000 China; 2 School of Information and Software Engineering, University of Electronic Science and Technology of China, Chengdu, 610054 China

**Keywords:** Computer science, Information technology

## Abstract

Generative adversarial networks have achieved strong results in computer vision, but their use in time series forecasting remains limited. This paper proposes a conditional noise generative adversarial network with a Siamese neural network as discriminator for long-term forecasting. The method combines the simplicity of a linear model with a generative framework, introducing a triplet margin loss to capture relationships between samples and conditional noise to improve sample generation. Experiments on eight open-source datasets show an average improvement of 8.42 percent, and a 192.8 percent gain for longer-term forecasting, with further improvement on a real-world telecommunications dataset.

## Introduction

Time series forecasting has a wide range of applications across various fields, including traffic flow prediction, weather forecastinging, disease transmission, and power equipment status monitoring and so on. Over the past few decades, time series forecasting methods have evolved significantly. Traditional statistical methods, such as Autoregressive Integrated Moving Average Model^1^ (ARIMA), have been foundational. This evolution continued with machine learning approaches like Gradient Boosting Regression Tree^2^ (GBRT) and advanced to neural network-based methods, including Long Short-Term Memory^3^ (LSTM) and Temporal Convolutional Network^4^ (TCN).

Neural network based methods for time series forecasting can be broadly categorized into Recurrent Neural Network^5^ (RNN) methods and non-RNN methods^6–9^. RNN is a neural network for processing sequence data, with its recurrent structure capable of capturing short-term dependency temporal information. However, RNN often face issues like gradient explosion when dealing with long-term temporal dependencies. Long Short-Term Memory (LSTM) network, a specialized type of RNN, address long-term dependency problems through a sophisticated gate mechanism. Gated Recurrent Units^10^ (GRU) are another variant of RNN, designed to simplify LSTM structures while retaining their ability to capture long-term dependencies. GRU combine the input gates and forget gates of LSTM into two main gates: the update gate and the reset gate, resulting in fewer parameters and higher computational efficiency. Despite their effectiveness, methods based on gating mechanisms, such as LSTM and GRU, come with increased computational complexity and lengthy training processes. These methods are still susceptible to gradient vanishing and exploding issues when processing long sequences. Additionally, their autoregressive nature complicates the parallelization of calculations.

Temporal Convolutional Network (TCN) is a new approach for processing sequence data using convolutional neural networks. This study has shown that convolutional architectures can outperform recurrent neural network in certain tasks. LogTrans^11^ introduced a convolutional self-attention mechanism and a sparse Transformer to address the challenges of capturing local context information and the memory bottleneck in long-term time series forecasting, making the Transformer architecture feasible for time series forecasting. Informer^12^ tackled the issues of Transformer’s time complexity and sensitivity to sequence length with a probabilistic sparse self-attention mechanism. Autoformer^13^ introduced the concept of decomposing time series into seasonal and trend-cyclical data. Its decomposition module, combined with an auto-correlation module, offers greater advantages than the traditional self-attention module. Pyraformer^14^ proposed replacing the self-attention mechanism with a resolution pyramid-based approach to extract time dependencies across different ranges. FEDformer^15^ further enhanced long-term time series forecasting by utilizing Fourier transform in the frequency domain to calculate self-attention.

Time series forecasting methods based on the Transformer architecture primarily aim to enhance the effectiveness and efficiency of capturing contextual information by replacing or developing the self-attention mechanism. DLinear^16^ questioned the effectiveness of using the Transformer architecture for long time series forecasting. The single-layer linear model it proposed significantly outperformed more complex Transformer-based models. Based on DLinear, TSMixer^17^ introduced a mixed time series forecasting model based on MLP. TSMixer effectively extracts feature information through mixed operations on the time and feature dimensions, emphasizing the importance of incorporating cross-variable and auxiliary information in long time series forecasting tasks.

The Siamese Neural Network^18^ (SNN) is a specialized neural network architecture primarily used for comparing the similarity between two inputs. It is proposed in 1993, SNN has been widely applied in tasks such as signature verification. SNN has two or more sub-networks with shared weights, which judge the similarity of inputs by calculating the distance between their feature representations. Leveraging the characteristics of SNN, this study designed a discriminator based on the SNN which naturally models the adversarial loss within generative adversarial network.

## Related works

Generative Adversarial Network^19^ (GAN) are a neural network-based method with the core idea of generating high-quality data that closely resembles real data through an adversarial process between two neural networks: the generator and the discriminator. GAN have a wide range of applications, including image generation, image super-resolution, data augmentation, and text generation. TimeGAN^20^ introduced a framework that combines unsupervised adversarial training with supervised learning to make the training process more controllable. This framework separates the input into static features and time series features. Initially, an encoder-decoder is trained in a latent space to restore predicted data. Then, a GAN is trained within the same latent space. The generator first undergoes supervised learning with the encoder in the latent space, followed by the decoder restoring the predicted results. Finally, adversarial training is conducted using the discriminator. This framework employs three loss functions for joint training, making the hyperparameters challenging to tune and the training difficult to converge. SocialGAN^21^ proposed using GAN to forecast pedestrian trajectory sequence. The generator in SocialGAN uses an encoder-decoder architecture with LSTM and pooling modules, while the discriminator, also composed of LSTM, measures the social acceptability of real and generated trajectories. SeqGAN^22^ addressed the challenge of generating discrete sequences. Given an input sequence, the generator creates a target sequence from all candidate tokens using an LSTM-based recurrent neural network, while the discriminator is a convolutional neural network. Innovatively, SeqGAN treats the discriminator as a reward model for reinforcement learning, updating the expected reward through Monte Carlo search.

TimeGAN employs a latent space embedding and recovery mechanism, reconstructing entire time series sequences. It samples random noise inputs into an encoder for generative prediction, which may not fully capture temporal dependencies. In contrast, CNGAN utilizes a linear encoder to generate conditionally noise directly, eliminating the need for additional random noise inputs and encoding steps. This approach enhances the model’s ability to capture temporal information more effectively. SocialGAN features a typical GAN architecture with an LSTM-based encoder-decoder generator and an LSTM discriminator. It generates sequences based solely on historical time series data. However, CNGAN encodes both historical and target prediction data in a single step, providing a richer information context for generation. SeqGAN integrates GANs with reinforcement learning, modeling sequence generation as a reinforcement learning task. The generator acts as a policy, and the discriminator provides reward signals. While innovative, SeqGAN does not leverage the rich information present in historical data, particularly in time series with periodic characteristics.

Recent studies have demonstrated the effectiveness of GANs in time series forecasting and anomaly detection. For instance, the Multi-attention GAN model^23^ for multi-step vegetation index forecasting improves accuracy by capturing long-term temporal patterns. Conditional Wasserstein GAN with Gradient Penalty (CWGAN-GP)^24^ has been proposed to generate synthetic time-series data to overcome data scarcity, enhancing demand prediction for electric vehicles. Additionally, the Predictive Wasserstein GAN with Gradient Penalty (PW-GAN-GP)^25^ has been introduced for anomaly detection in cloud data centers, outperforming baseline models in precision and recall. These approaches highlight the potential of GANs to enhance time series forecasting tasks, aligning with the methodology presented in our work.

Over the past few decades, GAN have achieved significant success in fields such as computer vision, image style transfer, and data augmentation. Numerous studies have demonstrated the potential of GAN to generate desired content from noise. The primary goal of most GAN-based methods is to create simulated data from noise.

This study explores, for the first time, a generative long-term time series forecasting method based on GAN, showcasing the potential of GAN in generating time series forecasting sequences. It is demonstrated that the adversarial training process inherent in GAN can produce forecasting sequences that closely align with the real data distribution, resulting in more robust predictions and improved model generalization. It significantly increases the difficulty of the generator generative prediction because completely random noise is not suitable for time series forecasting which has specific temporal information. To address this, conditional noise is designed and pre-trained that incorporates posterior information to reduces the complexity of the generator generative prediction process. GAN are expected to become a crucial tool in long-term time series forecasting, enabling researchers and practitioners to capture complex temporal patterns more accurately, thereby facilitating more informed decision-making.

The main contributions of this study are:

1. This paper proposed parameterized conditional noise and optimized its parameters through supervised pre-training, transforming it from random noise into conditional noise with posterior information which can enhance the quality of the generated prediction.
2. This paper introduced the Conditional Noise Generative Adversarial Network (CNGAN) and explored the integration of SNN with GAN. By leveraging the features of SNN and GANs, “real” samples and generated “fake” samples are feed into the SNN in pairs to calculate the triplet margin loss. This architecture naturally models the adversarial nature of GAN which can enable forecasting sequences closely align with the real data distribution, resulting in more robust predictions and improved model generalization.
3. This paper conducted extensive experiments on the opensource datasets and Jiangsu Telecom market datasets. The results demonstrate that the proposed method shows significant advancement and adaptability for sparse long-term time series forecasting.

## Datasets and definition

As shown in Table 1, for the open-source time series forecasting dataset, time series data is a series of feature vectors that depend on time *t*, expressed as $X_{1:L}=[x_1,...,x_L]^T\in \mathbb {R}^{L\times d}$. Here, $L$ represents the total steps, $x_t\in \mathbb {R}^{1\times d}$ represents the feature vector at time $t$. The task of time series forecasting is fitting conditional distribution $p(X_{t:t+T-1}|X_{t-h:t-1})$. Here, $T$ represents the forecast steps, $h$ represents the history series length. The task aims to capture the conditional distribution of history time series to forecast subsequent time series. When $T=1$, it indicates a single-step forecast, and when $T>1$, it indicates a multi-steps forecast. Multi-steps forecast can be further divided into long-term and short-term predictions. The prediction can be either univariable or multivariable. This paper focuses on long-term multivariable forecasting.

**Table 1:** Statistics of datasets.

Dataset	Open-source	Number of input variables(*d*)	Total steps(*L*)	Graininess
ETTh1/h2	$\checkmark$	7	17420	1hour
ETTm1/m2	$\checkmark$	7	699680	5min
National illness	$\checkmark$	7	966	1week
Weather	$\checkmark$	21	52696	10min
Exchange Rate	$\checkmark$	8	7588	1day
Electricity	$\checkmark$	321	26304	1hour
Traffic	$\checkmark$	862	17544	1hour
Telecom market	✗	25	14	1month

Generally, time series forecasting use mean squared error (MSE) and mean absolute error (MAE) as evaluation metrics. These metrics are maintained for the open-source datasets, ETT and National illness.

For the closed-source Telecom market dataset, the data structure is different. This paper collected 14 months of marketing data from January 2023 to February 2024, with a collection granularity of one month. The original data is stored in a large-scale Hive data warehouse, distributed across multiple large tables. First, several hundred lines of HQL code are executed to extract user data for 14 months from the Hive database. Subsequently, it is necessary to filter users based on specific information, such as age, fees, and the products. After that, N/A and outliers values are filtered out, followed by embedding processing for discrete features and temporal information. Finally, the focus is placed on the target feature, which indicates whether a specific product has been subscribed (True or False). After this preprocessing, the final dataset includes 87,650 existing users (where existing users refer to those who were not new as of January 2023). The objective is to predict the propensity of users who have not currently subscribed to a specific product to do so in the future, based on their history data.

For each customer, 26 user features were collected, including network traffic, behavioral preferences, etc. Consequently, this dataset includes an additional user quantity dimension compared to the open-source time series forecasting datasets. The datasets can be expressed as $X_{1:14}=[x_1,...,x_{14}]$. Here $X\in \mathbb {R}^{N\times 14\times 26}$ represents all user data for the 14 months, $x_t=[u_{t,1},...u_{t,N}]^T\in \mathbb {R}^{N\times 1 \times 26}$ denotes all user data for $t$-month, and $u_{t,i}\in \mathbb {R}^{1\times 26}$ is the 26 features of $i$-user at $t$-month.

According to business needs, the dataset is divided into a training set (from January 2023 to December 2023) and a test set (from January 2024 to February 2024). The task for the training set is to perform single-step forecasting based on the different input sequence length, it can be expressed as $p(X_t|X_{1:t-1},t\in [2,12])$.It means that when the input sequence length is 1 to 11, a single-step forecasting (multivariate to univariable) is performed, and the target variable is a $label=\{0,1\}$.

For the closed-source Telecom market dataset, the evaluation metrics differ from the ETT and National illness. Since marketing units typically need to define a fixed number of marketing targets, this study sorts the output probability and selects the top 5%, 10%, and 30% of the user groups as marketing targets (considered as positive). The binary classification metrics, including precision, recall, and F1-score, are then calculated for evaluation. The main difference is the sparsity. Specifically, the sampling step of this dataset is one month, while the sampling interval of most other open source datasets is usually less than one hour. This longer sampling interval makes the data more sparse in the time dimension, and the frequency of information acquisition is lower. So it may face higher challenges. The experimental results in Section 6 show that for sparse dataset, CNGAN we proposed shows significant performance improvements in both accuracy and robustness, especially when processing data with longer time intervals.

## Method

### Architecture

**Fig. 1:**
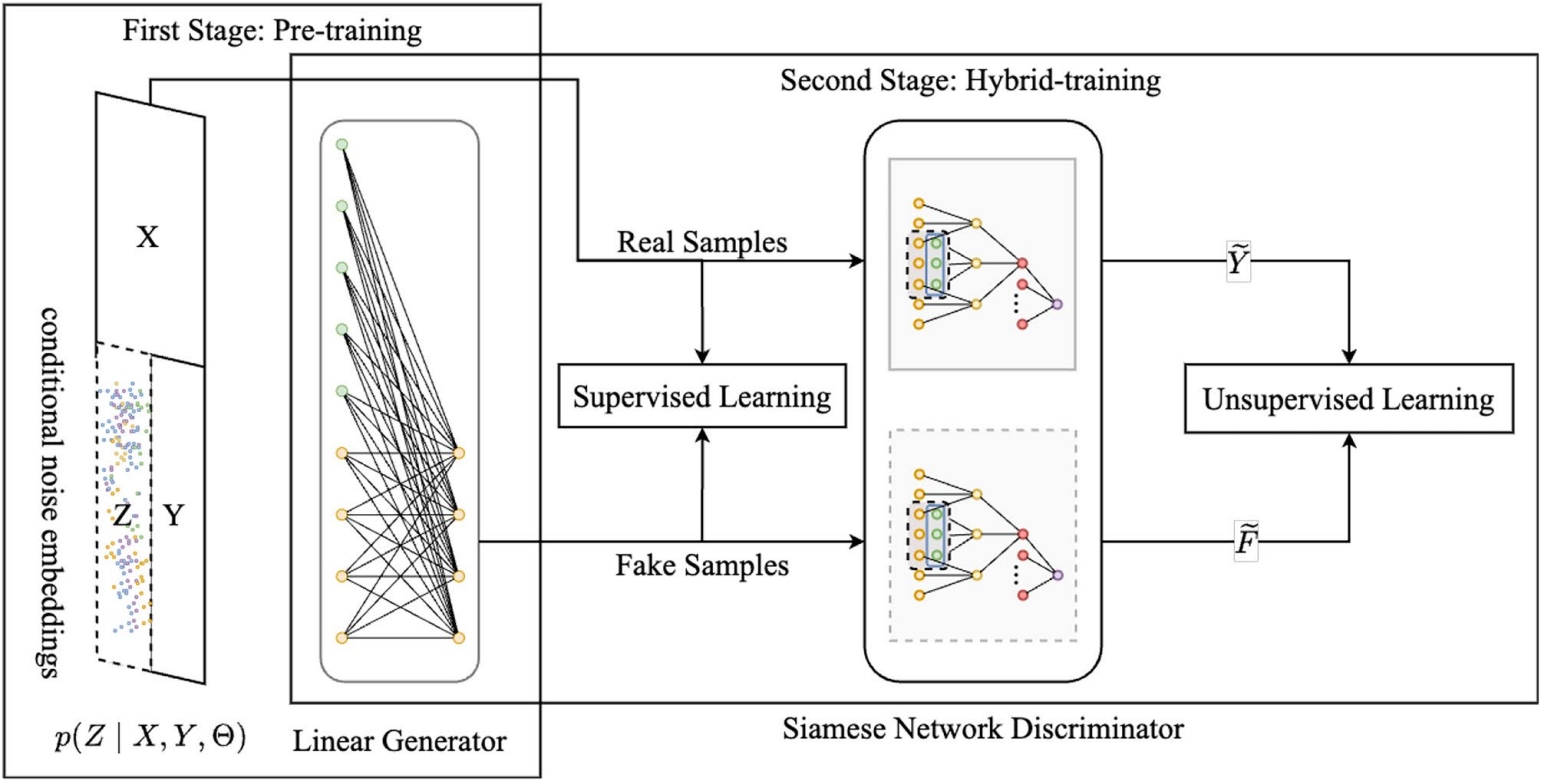
Architecture of CNGAN. The training process is divided into two stages. In the first stage, the conditional noise embeddings and its generator are pre-trained in a supervised manner. In the second stage, the generative adversarial network is trained using a joint loss that combines supervised and unsupervised learning.

This paper proposes a novel long-term time series forecasting method based on conditional noise and generative adversarial networks (Conditional Noise Generative Adversarial Networks, CNGAN). The overall architecture of CNGAN is shown in Fig. 1. It consists of three main components: conditional noise generator, SNN discriminator and Linear Generator.

The training process is divided into two stages. In the first stage, the conditional noise embeddings and its generator are pre-trained in a supervised manner. In the second stage, the generative adversarial network is trained using a joint loss that combines supervised and unsupervised learning. This architecture can incorporate valuable additional information, including posterior conditional noise that encodes the complete input sequence information in the latent space, supervised learning information for time series forecasting, and unsupervised adversarial information).

### Conditional noise

Generation of synthetic samples by tradition GAN from completely random noise causes deficiency in the quality and diversity since the unutilization of the posterior knowledge. This paper introduces posterior knowledge to random noise, incorporating more contextual information into the generation process to enhance the quality of the generated data. Random initialization $Z\in \mathbb {R}^{L\times d}$ represents a non-informative prior. The pretrained embedding and generator $p(Z,\Theta |X,Y)$ can be viewed as a posterior representation and an encoder derived from the pretraining data, respectively.

**Fig. 2:**
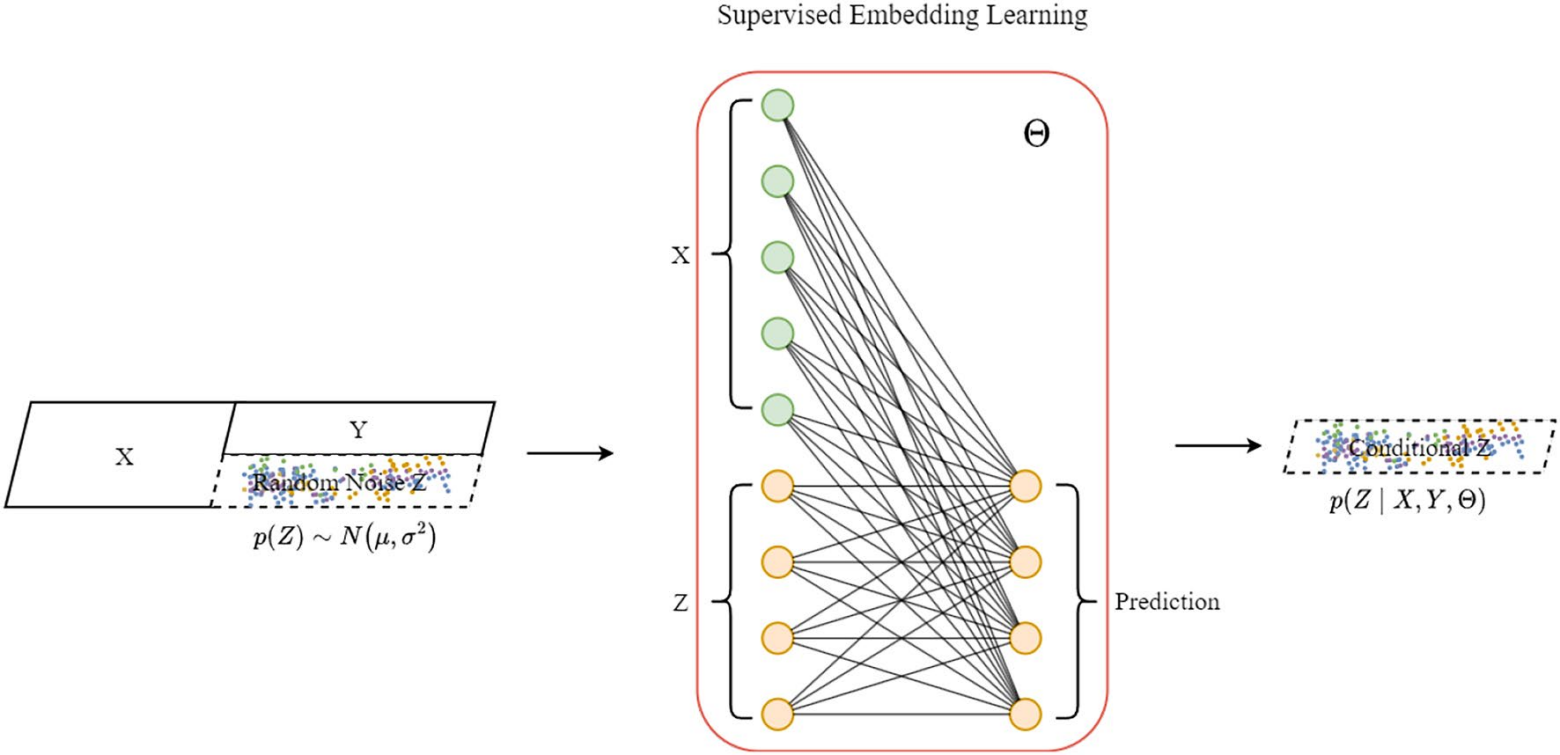
Conditional noise. The model takes the historical sequence *X* and conditional noise *Z* as inputs, and generates the predicted sequence $\tilde{Y}$. The object is minimizing the mean squared error (MSE) loss between the ground-truth sequence *Y* and the predicted sequence $\tilde{Y}$, thereby optimizing the conditional noise embedding *Z*.

In the pretraining stage, conditional information derived from the data distribution and target labels is incorporated, enabling the learned conditional noise to carry posterior knowledge such as class-specific features and structural patterns of the target data. As a result, the generator receives more informed and goal-directed inputs rather than relying solely on random exploration. This posterior-guided input enhances the contextual consistency and realism of the generated samples. Additionally, this approach encourages the generator to learn a wider variety of data combination patterns, making the generated data more representative of the actual distribution and reducing the likelihood of mode collapse. As shown in Fig. 2, this study uses a linear layer as the conditional noise generator. The conditional noise embeddings are initialized using a Gaussian distribution as Eq. (1):

1 $$\begin{aligned} Initiate(Z)=p(Z)\sim \mathcal {N}(\mu ,\sigma ^2) \end{aligned}$$

During pre-training, both the embeddings $Z\in \mathbb {R}^{L\times d}$ and the conditional noise generator $\Theta \in \mathbb {R}^{(l+L)\times L}$ are optimized.

The parameterized *Z* and $\Theta$ is optimized to minimize the $loss_c$ as Eq. (2):

2 $$\begin{aligned} \underset{Z,\Theta }{\min }~loss_c(Z,\Theta )=\frac{1}{n}\sum _{i=1}^n(y_i-[x_i;Z]^T\cdot \Theta )^2 \end{aligned}$$

which supervised pre-training process can be expressed as Eq. (3)(4):

3 $$\begin{aligned}&\nabla loss_c(Z,\Theta )=(\frac{\partial loss_c}{\partial Z},\frac{\partial loss_c}{\partial \Theta })^T \end{aligned}$$

4 $$\begin{aligned}&(Z,\Theta )^T\leftarrow (Z,\Theta )^T-\eta \nabla loss_c(Z,\Theta ) \end{aligned}$$

Here, $\eta$ represents the learning rate of the noise embedding model, *y* represents the target vector, *n* represents the dimensions of features. Through this supervised pre-training process, we obtains a conditional noise embedding and corresponding generator that encode complete sequence information as Eq. (5):

5 $$\begin{aligned} Conditional Noise = p(Z|X,Y,\Theta ) \end{aligned}$$

### Generative adversarial networks

#### SNN discriminator

**Fig. 3:**
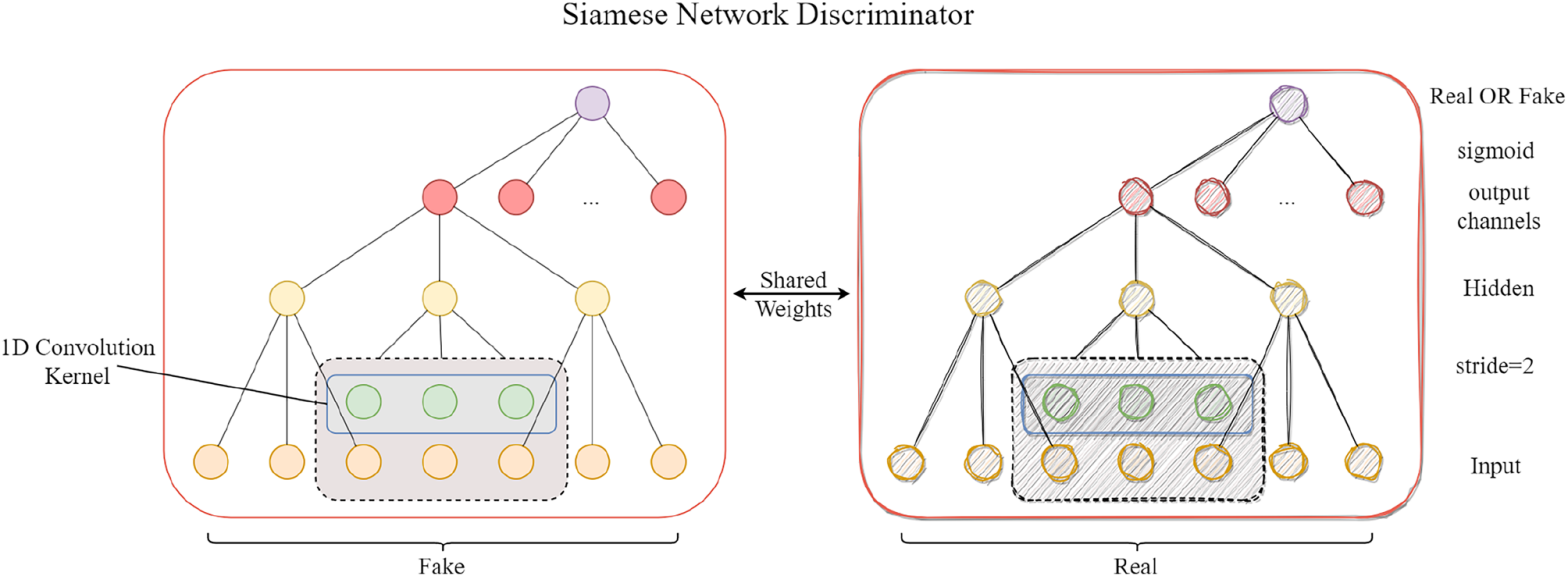
SNN Discriminator. the SNN discriminator consists of two sub-networks with the same structure and shared parameters.

As shown in Fig. 3, the SNN discriminator consists of two sub-networks with the same structure and shared parameters. In this study, each sub-network of the SNN employs a multi-layer one-dimensional convolutional neural network. One-dimensional convolution is effective for processing time series data, capturing time series features at various resolutions along the sequence dimension.

The adoption of a Siamese network is a natural and intuitive choice, owing to its inherent ability to process sample pairs in parallel. This architectural characteristic aligns seamlessly with the use of a triplet loss function, enabling the model to naturally capture the relative relationships between real and synthetic samples, rather than making independent and absolute judgments about their authenticity.

Each sub-network first processes the input data through a series of one-dimensional convolutional layers. The convolutional operation extracts local features through a sliding window mechanism, retaining the overall structure of the time series. Finally, a fully connected layer integrates these features across the variable dimension. The output is a scalar value mapping the features to a low-dimensional space. Assuming the input data is $X\in \mathbb {R}^{d\times (l+L)}$, the forward process can be expressed as Eq. (6):

6 $$\begin{aligned} f(X,W_1,W_2)=Sigmoid(FC(CNN(X,W_1),W_2))\in (0,1) \end{aligned}$$

**Fig. 4:**
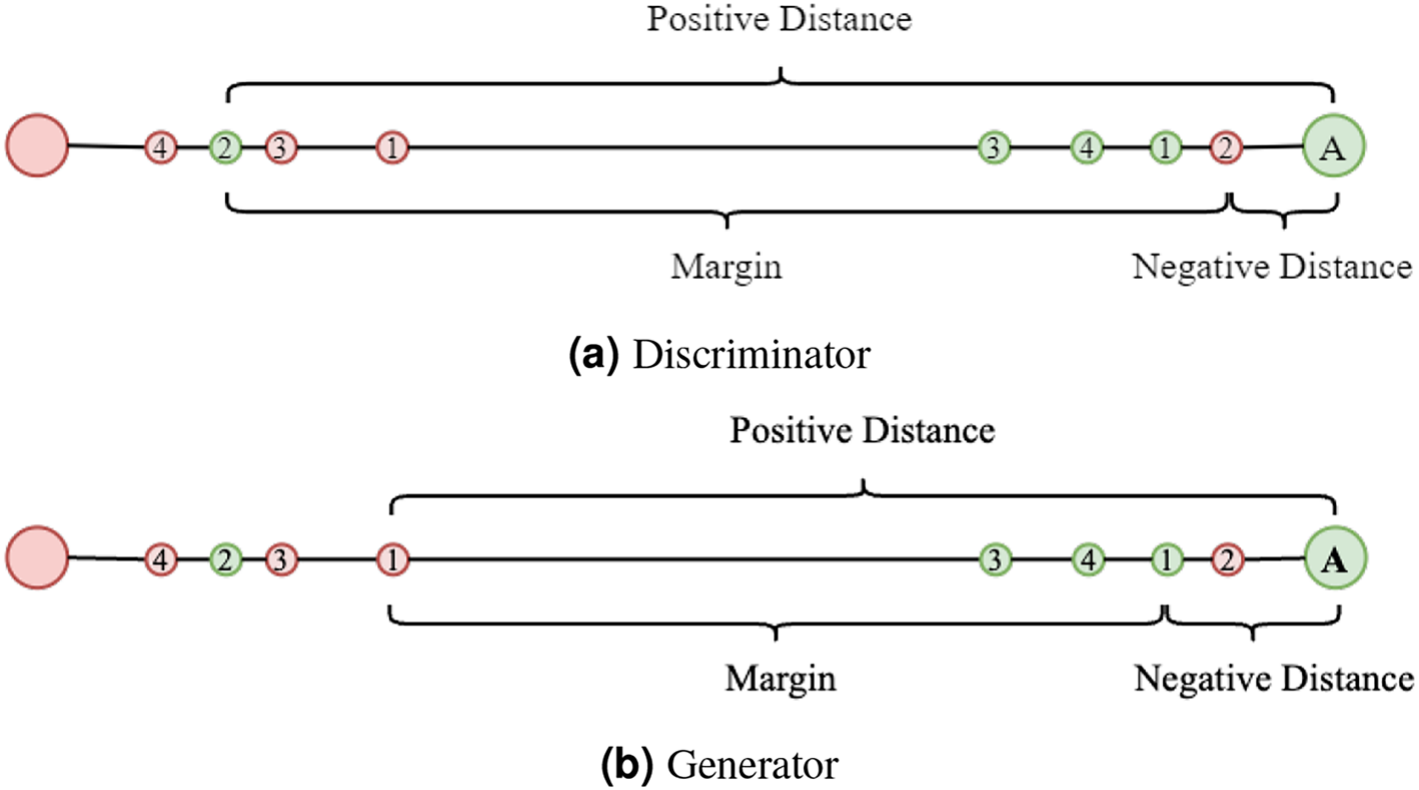
Triplet margin of discriminator and generator. Naturally inputs paired real and fake data into the SNN discriminator and calculates the triplet margin loss relative to an “Anchor”.

Here, FC represents the Full-Connected Layer, CNN represents 1D-Convolution, $W_1$ and $W_2$ represent the parameter of CNN and FC, X is inputs. Intuitively, as shown in Fig. 4a, this study naturally inputs paired real and fake data into the SNN discriminator and calculates the triplet margin loss relative to an “Anchor”.

As shown in the Eq. (7), the task of the discriminator is to minimize the distance of the real data relative to the Anchor and maximize the distance of the fake data relative to the Anchor. For instance, in the case of real sample 2 and fake sample 2 in Fig. 4a, the SNN discriminator aims to minimize the positive distance and simultaneously maximize the negative distance. This objective can be reformulated as minimizing the difference between these two distances, ultimately yielding a discriminator capable of effectively distinguishing between real and fake samples. The triplet margin loss enables the network to learn the relative relationships between real and fake samples, rather than focusing on absolute classification information.

The objective of discriminator training is to minimize the $loss_D$ by optimizing the parameters $W_1$ and $W_2$ as Eq. (7):

7 $$\begin{aligned} \underset{W_1,W_2}{\min }~loss_D(W_1,W_2)=\max \{(A-f(X,W_1,W_2))^2-(A-f(\widetilde{X},W_1,W_2))^2+m,0\} \end{aligned}$$

which training process can be expressed as Eq. (8)(9):

8 $$\begin{aligned}&\nabla loss_D(W_1,W_2)=(\frac{\partial f_{X}}{\partial W_1}-\frac{\partial f_{\widetilde{X}}}{\partial W_1},\frac{\partial f_{X}}{\partial W_2}-\frac{\partial f_{\widetilde{X}}}{\partial W_2})^T \end{aligned}$$

9 $$\begin{aligned}&(W_1,W_2)^T\leftarrow (W_1,W_2)^T-\epsilon \nabla loss_D(W_1,W_2) \end{aligned}$$

Here, $X$ and $\widetilde{X}$ represent real samples and fake samples generated by the generator, respectively. Hyper-parameter $m$ is defined as the minimum separation that should exist between the distance of the anchor–positive pair and the anchor–negative pair. The margin enforces that the negative sample must be at least margin units farther from the anchor than the positive sample. A larger margin imposes a stricter constraint, encouraging greater inter-class separation but potentially making optimization more difficult, while a smaller margin relaxes the constraint, possibly leading to faster convergence but weaker feature discrimination. $\epsilon$ represents the learning rate for training discriminator.

#### Linear generator

**Fig. 5:**
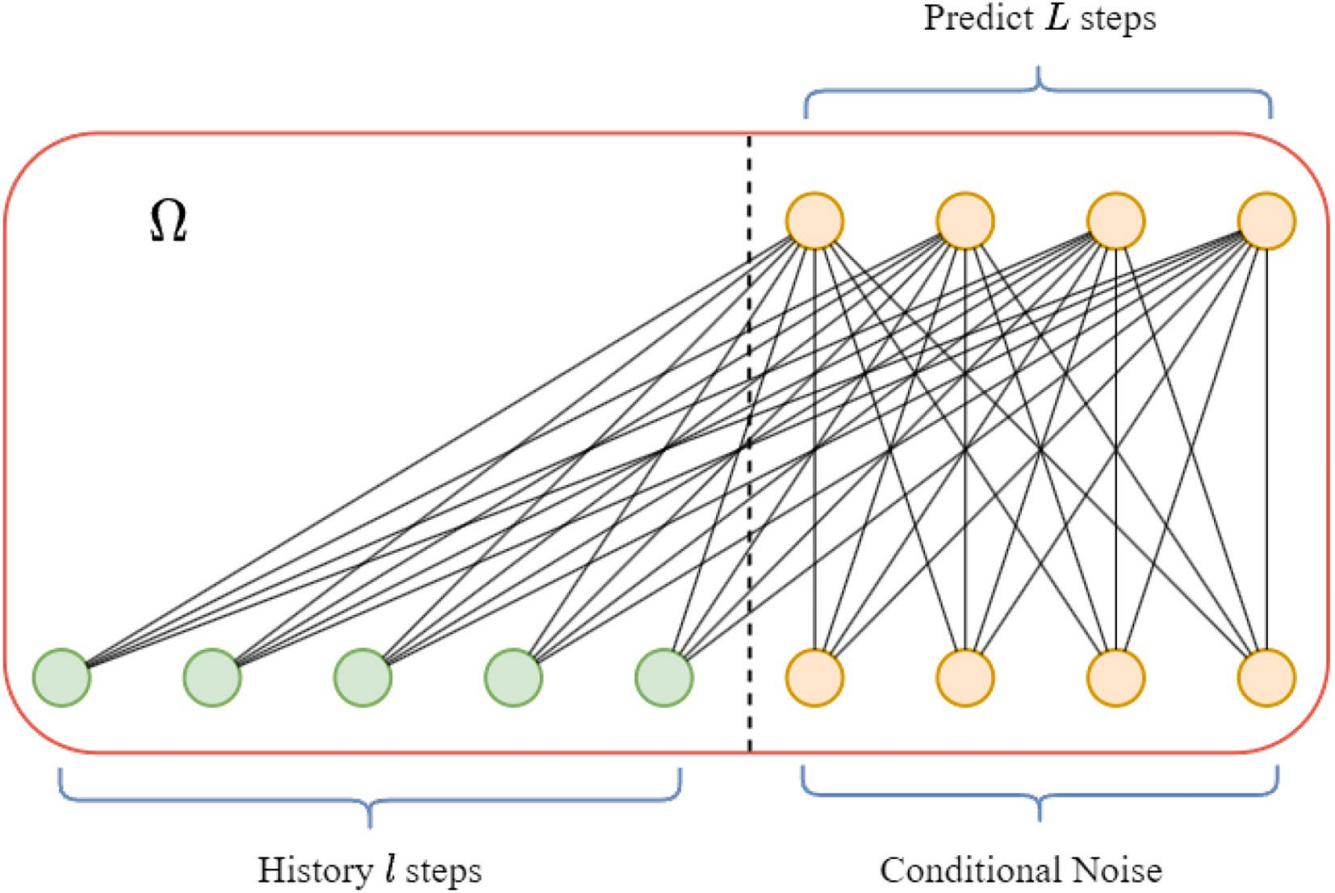
Linear generator. Serves as the generator in the GAN architecture.

The work of paper^16^ has demonstrated that using a simple linear model can achieve competitive results in long-term time series forecasting. As shown in Fig. 5, this study employs a linear model similar to the noise generator as the generator of GAN. The difference from ^16^ lies in the addition of conditional noise to the input sequence as information with posterior knowledge. This enhancement helps the generator capture more contextual information and temporal patterns, thereby improving the quality of generated data and preventing pattern collapse.

The input to the generator is the concatenation of historical sequence $H\in \mathbb {R}^{l\times d}$ and conditional noise $Z\in \mathbb {R}^{L\times d}$, and its output is the forecasting result for $L$ steps $p(X_{t:t+T-1}|X_{t-l:t-1})$. The goal of the generator is to make the prediction result as “accuracy” and “real” as possible. Here, “accuracy” refers to minimizing the mean squared error loss between the predicted data and the ground truth, while “real” refers to minimizing the triplet margin loss calculated using the SNN discriminator. As shown in Fig. 4b, the adversarial loss during the training of the generator aims to minimize fake “positive” distance and real “negative” distance. As shown in the Eq. (10), this objective is also transformed into minimizing the difference between two distances.

The objective of generator training is to minimize the unsupervised $g(\Omega )$ by optimizing the generator parameters $\Omega$ and supervised mse loss with hyper-parameter $\alpha$ as Eq. (10):

10 $$\begin{aligned} \underset{\Omega }{\min }~loss_G=\alpha g(\Omega )+(1-\alpha )(Y-[H;Z]^T\cdot \Omega )^2 \end{aligned}$$

where $g_(\Omega )$ is expressed as:

11 $$\begin{aligned} \underset{\Omega }{\min }~g(\Omega )=\max \{(A-f([H;Z]^T\cdot \Omega ,W_1,W_2))^2-(A-f(X,W_1,W_2))^2+m,0\} \end{aligned}$$

Then the training process can be expressed as Eq. (12)(13):

12 $$\begin{aligned}&\nabla loss_G(\Omega )=\alpha \frac{\partial g}{\partial f}\frac{\partial f}{\partial \Omega }-2(1-\alpha )[H;Z]^T(Y-[H;Z]^T\cdot \Omega ) \end{aligned}$$

13 $$\begin{aligned}&\Omega \leftarrow \Omega -\mu \nabla loss_G(\Omega ) \end{aligned}$$

Here, $\alpha$ is a hyper-parameter that controls the strength of the adversarial loss, and $\mu$ is the learning rate for training generator. The loss of the generator consists of two parts: one is the supervised loss that measures “accuracy”, and the other is the unsupervised loss that measures “authenticity”. This method introduces valuable supervisory information, and the unsupervised loss utilizes the gradient of the discriminator $\frac{\partial f}{\partial \Omega }$ to optimize the parameters of the generator. This approach allows the entire network to simultaneously optimize both “accuracy” and “authenticity”. By sharing information between two tasks, the model enhances its generalized ability and improves overall performance.

## Main results

This study conducted extensive experiments on five open-source time series forecasting datasets using the following hardware environments: 12th Gen Intel(R) Core(TM) i5-12600KF 3.69 GHz processor, NVIDIA GeForce RTX 3070 Ti 8G graphics card, and 32 GB of memory. Additionally, the study collected a closed-source Telecom market dataset from Jiangsu Telecom’s database for further experiments, using Intel(R) Xeon(R) Gold 5220 CPU @ 2.20 GHz processor, NVIDIA Tesla T4 16G graphics card, and 32 GB of memory. The Experiment settings are presented in Table 2.

**Table 2:** Experiment settings.

Task	Dataset	Metric	Steps of input	Steps of forecast
Time series forecast	ETTh1/h2, ETTm1/m2	MAE, MSE	512	96$\sim$720
	National illness	MAE, MSE	104	24$\sim$60
	Weather	MAE, MSE	512	96$\sim$720
	Exchange Rate	MAE, MSE	512	96$\sim$720
	Electricity	MAE, MSE	512	96$\sim$720
	Traffic	MAE, MSE	512	96$\sim$720
Classification	Telecom market	Precision, Recall, F1-score	1$\sim$11	1

### Results on open-source datasets

The results on the open-source datasets are shown in Table 3. This study compared the linear methods and five Transformer-based methods. All results marked with * are from the paper ^16^. The best results are indicated in black, and the second best are underlined. CNGAN is the method proposed in this study. The simple but SOTA linear methods, Linear and DLinear, showed improvements ranging from 20% to 50% compared to the Transformer-based methods. CNGAN improved totally by 138.3%(The sum of the IMP. in Table 3. IMP. quantifies the performance gain of CNGAN by summing the differences in MSE and MAE metrics relative to other baseline methods.) compared with DLinear on all open-source datasets.

Moreover, compared to other GAN-based methods, such as TimeGAN, TimeGAN was unable to achieve competitive performance compared to the proposed CNGAN method. TimeGAN employs a latent space embedding and recovery mechanism, reconstructing entire time series sequences. It relies on purely random noise as input to the encoder for generative prediction, without incorporating pretrained knowledge or prior information, which may limit its ability to fully capture temporal dependencies. In contrast, CNGAN utilizes a linear encoder to generate conditional noise directly, leveraging pretrained information and eliminating the need for additional random noise inputs and encoding steps. This approach enhances the model’s ability to capture temporal patterns more effectively.

**Table 3:** Results for multivariate time series forecasting.

Methods			Linear*		DLinear*		TimeGAN		CNGAN			
Datasets	Steps	IMP. (%)	MSE	MAE	MSE	MAE	MSE	MAE	MSE	MAE		
ETTh1	96	1.5	0.375	0.397	0.375	0.399	0.399	0.420	**0.368**	**0.391**		
	192	0.7	0.418	0.429	0.405	0.416	0.415	0.423	**0.402**	**0.412**		
	336	2.8	0.479	0.476	0.439	0.443	0.450	0.464	**0.426**	**0.428**		
	720	5.7	0.624	0.592	0.472	0.490	0.497	0.504	**0.444**	**0.461**		
ETTh2	96	1.9	0.288	0.352	0.289	0.353	0.304	0.387	**0.279**	**0.344**		
	192	4.8	0.377	0.413	0.383	0.418	0.388	0.421	**0.358**	**0.395**		
	336	8.2	0.452	0.461	0.448	0.465	0.459	0.488	**0.404**	**0.427**		
	720	19.6	0.698	0.595	0.605	0.551	0.511	0.554	0.477	0.483		
ETTm1	96	0.1	0.308	0.352	0.299	0.343	0.332	0.369	0.300	**0.341**		
	192	0.2	0.340	0.369	0.335	0.365	0.353	0.385	**0.334**	**0.364**		
	336	0.0	0.376	0.393	**0.369**	0.386	0.395	0.402	0.370	**0.385**		
	720	1.1	0.440	0.435	0.425	0.421	0.437	0.443	**0.421**	**0.414**		
ETTm2	96	0.7	0.168	0.262	0.167	0.260	0.202	0.294	**0.165**	**0.255**		
	192	0.1	0.232	0.308	0.224	0.303	0.254	0.313	0.225	**0.301**		
	336	1.0	0.320	0.373	0.281	0.342	0.317	0.359	**0.276**	**0.337**		
	720	2.1	0.413	0.435	0.397	0.421	0.418	0.444	**0.389**	**0.408**		
ILI	24	32.6	1.947	0.985	2.215	1.081	2.164	1.418	2.051	**0.919**		
	36	32.6	2.182	1.036	1.963	0.963	2.034	1.294	**1.737**	**0.863**		
	48	59.7	2.256	1.060	2.130	1.024	2.318	1.116	**1.701**	**0.856**		
	60	82.6	2.390	1.104	2.368	1.096	2.446	1.193	**1.744**	**0.894**		
Weather	96	2.4	0.176	0.236	0.176	0.237	0.193	0.255	**0.168**	**0.221**		
	192	2.7	0.218	0.276	0.220	0.282	0.231	0.281	**0.214**	**0.261**		
	336	2.8	0.262	0.312	0.265	0.319	0.284	0.337	**0.257**	**0.299**		
	720	0.0	0.326	0.365	**0.323**	0.362	0.342	0.379	0.329	**0.356**		
Exchange Rate	96	− 0.9	0.082	0.207	**0.081**	**0.203**	0.125	0.281	0.085	0.208		
	192	− 1.3	0.167	0.304	**0.157**	**0.293**	0.270	0.345	0.164	0.299		
	336	1.1	0.328	0.432	0.305	**0.414**	0.316	0.462	**0.293**	0.415		
	720	6.3	0.964	0.750	0.643	0.601	0.593	0.674	**0.586**	**0.595**		
Electricity	96	0.8	0.140	0.237	0.140	0.237	0.164	0.276	**0.136**	**0.233**		
	192	0.4	0.153	0.250	0.153	0.249	0.182	0.283	**0.151**	**0.247**		
	336	0.3	0.169	0.268	0.169	0.267	0.251	0.297	**0.167**	**0.266**		
	720	− 0.4	**0.203**	**0.301**	**0.203**	**0.301**	0.247	0.332	0.206	0.302		
Traffic	96	− 4.7	**0.410**	**0.282**	**0.410**	**0.282**	0.455	0.332	0.422	0.317		
	192	− 4.6	**0.423**	**0.287**	**0.423**	**0.287**	0.464	0.378	0.434	0.322		
	336	− 12.5	**0.436**	**0.295**	**0.436**	0.296	0.501	0.422	0.491	0.366		
	720	− 109.4	**0.466**	**0.315**	**0.466**	**0.315**	0.612	0.497	1.169	0.706		

Methods			FEDformer*		Autoformer*		Informer*		Pyraformer*		LogTrans*	
Datasets	Steps	IMP. (%)	MSE	MAE	MSE	MAE	MSE	MAE	MSE	MAE	MSE	MAE
ETTh1	96	1.5	0.376	0.419	0.449	0.459	0.865	0.713	0.664	0.612	0.878	0.740
	192	0.7	0.420	0.448	0.500	0.482	1.008	0.792	0.790	0.681	1.037	0.824
	336	2.8	0.459	0.465	0.521	0.496	1.107	0.809	0.891	0.738	1.238	0.932
	720	5.7	0.506	0.507	0.514	0.512	1.181	0.865	0.963	0.782	1.135	0.852
ETTh2	96	1.9	0.346	0.388	0.358	0.397	3.755	1.525	0.645	0.597	2.116	1.197
	192	4.8	0.429	0.439	0.456	0.452	5.602	1.931	0.788	0.683	4.315	1.635
	336	8.2	0.496	0.487	0.482	0.486	4.721	1.835	0.907	0.747	1.124	1.604
	720	19.6	**0.463**	**0.474**	0.515	0.511	3.647	1.625	0.963	0.783	3.188	1.540
ETTm1	96	0.1	0.379	0.419	0.505	0.475	0.672	0.571	0.543	0.510	0.600	0.546
	192	0.2	0.426	0.441	0.553	0.496	0.795	0.669	0.557	0.537	0.837	0.700
	336	0.0	0.445	0.459	0.621	0.537	1.212	0.871	0.754	0.655	1.124	0.832
	720	1.1	0.543	0.490	0.671	0.561	1.166	0.823	0.908	0.724	1.153	0.820
ETTm2	96	0.7	0.203	0.287	0.255	0.339	0.365	0.453	0.435	0.507	0.768	0.642
	192	0.1	0.269	0.328	0.281	0.340	0.533	0.563	0.730	0.673	0.989	0.757
	336	1.0	0.325	0.366	0.339	0.372	1.363	0.887	1.201	0.845	1.334	0.872
	720	2.1	0.421	0.415	0.433	0.432	3.379	1.338	3.625	1.451	3.048	1.328
ILI	24	32.6	3.228	1.260	3.483	1.287	5.764	1.677	**1.420**	2.012	4.480	1.444
	36	32.6	2.679	1.080	3.103	1.148	4.755	1.467	7.394	2.031	4.799	1.467
	48	59.7	2.622	1.078	2.669	1.085	4.763	1.469	7.551	2.057	4.800	1.468
	60	82.6	2.857	1.157	2.770	1.125	5.264	1.564	7.662	2.100	5.278	1.560
Weather	96	2.4	0.217	0.296	0.266	0.336	0.3	0.384	0.896	0.556	0.458	0.49
	192	2.7	0.276	0.336	0.307	0.367	0.598	0.544	0.622	0.624	0.658	0.589
	336	2.8	0.339	0.38	0.359	0.395	0.578	0.523	0.739	0.753	0.797	0.652
	720	0.0	0.403	0.428	0.419	0.428	1.059	0.741	1.004	0.934	0.869	0.675
Exchange Rate	96	− 0.9	0.193	0.308	0.201	0.317	0.274	0.368	0.386	0.449	0.258	0.357
	192	− 1.3	0.201	0.315	0.222	0.334	0.296	0.386	0.386	0.443	0.266	0.368
	336	1.1	0.214	0.329	0.231	0.338	0.3	0.394	0.378	0.443	0.28	0.38
	720	6.3	0.46	0.5	0.509	0.524	1.672	1.036	1.874	1.172	1.659	1.081
Electricity	96	0.8	1.195	0.841	1.447	0.941	2.478	1.31	1.943	1.206	1.941	1.127
	192	0.4	0.587	0.366	0.613	0.388	0.719	0.391	2.085	0.468	0.684	0.384
	336	0.3	0.246	0.355	0.254	0.361	0.373	0.439	0.376	0.445	0.283	0.376
	720	− 0.4	0.148	0.278	0.197	0.323	0.847	0.752	0.376	1.105	0.968	0.812
Traffic	96	− 4.7	0.271	0.38	0.3	0.369	1.204	0.895	1.748	1.151	1.04	0.851
	192	− 4.6	0.604	0.373	0.616	0.382	0.696	0.379	0.867	0.467	0.685	0.39
	336	− 12.5	0.621	0.383	0.622	0.337	0.777	0.42	0.869	0.469	0.734	0.408
	720	− 109.4	0.626	0.382	0.66	0.408	0.864	0.472	0.881	0.473	0.717	0.396

Bold and underlined text indicate the best and second-best performance, respectively.

Across all forecast step settings on the ETTh1/h2 and ETTm1/m2 datasets, CNGAN outperformed the DLinear method. The most significant improvement was observed when the forecast step was 720 on the ETTh2 dataset, achieving an MSE of 0.477 and MAE of 0.483, which was 19.6% higher than the comparison method. On the National illness dataset, improvement of the forecast performance ranges from 32% to 82%. However, it is poor on the other datasets. Even it shows a significant declines on Traffic dataset.

**Fig. 6:**
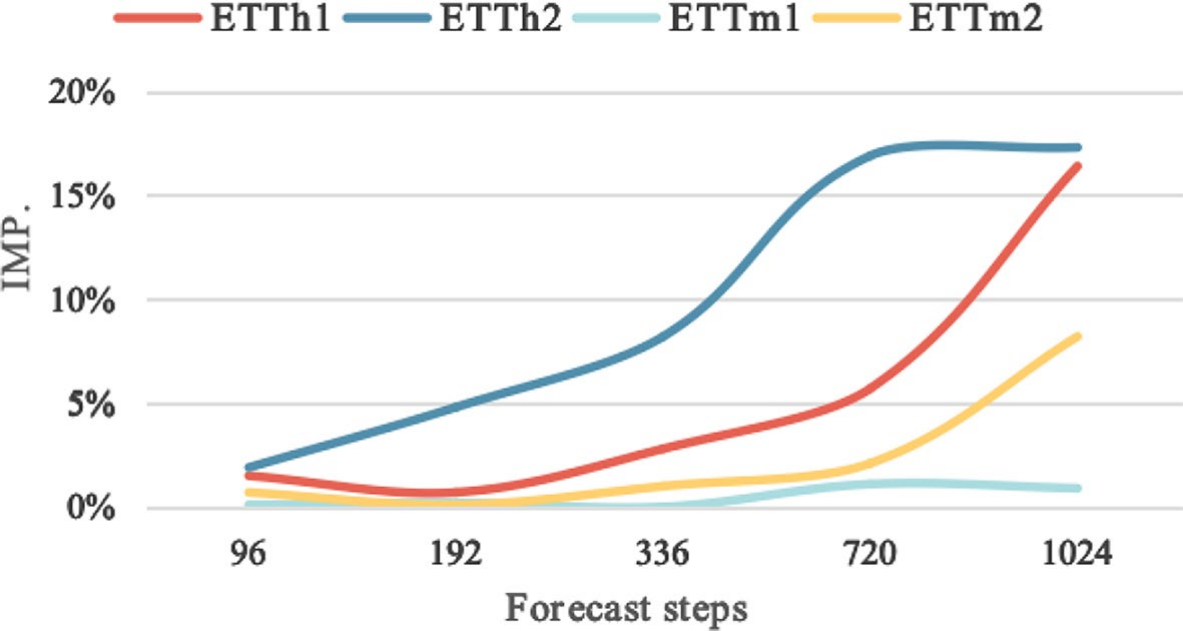
Improvement of different forecast steps. CNGAN surpasses both DLinear and Transformer-based methods, showing a gradually increasing trend in performance at longer forecast steps.

As shown in Fig. 6, the results demonstrate that CNGAN surpasses both DLinear and Transformer-based methods, showing a gradually increasing trend in performance at longer forecast steps.

As shown in Table 4, on five open source datasets, CNGAN achieved an impressive improvement of 192.8% for the longer forecast step settings of 1024 and 80. As the forecast steps increased, CNGAN significantly improved the forecast ability. However, there appears to be a shortcoming in shorter forecast steps.

**Table 4:** Results for longer steps on time series forecasting.

Methods			Linear		DLinear		CNGAN	
Datasets	Steps	IMP. (%)	MSE	MAE	MSE	MAE	MSE	MAE
ETTh1	1024	16.4	0.545	0.536	0.612	0.578	**0.519**	**0.507**
ETTh2	1024	17.3	0.804	0.634	0.754	0.616	**0.642**	**0.555**
ETTm1	1024	0.9	0.465	0.449	0.458	0.439	**0.454**	**0.434**
ETTm2	1024	8.2	0.438	0.438	0.493	0.473	**0.440**	**0.444**
ILI	80	150.0	2.673	1.175	2.963	1.283	**1.822**	**0.924**

Bold and underlined text indicate the best and second-best performance, respectively.

### Results on Jiangsu Telecom market dataset

This study also conducted experiments on Jiangsu Telecom’s closed-source market dataset, which possesses characteristics that differ from open source datasets: a short total step length, large granularity, and a user dimension in addition to time and feature dimensions. These differences collectively necessitate training with a fixed context window and sampling along the customer dimension, rather than sliding sampling across a longer sequence of data like open-source datasets. Shorter contexts also increase prediction difficulty. This experiment reports precision, recall, and F1-score as binary classification metrics. The model outputs the probability that a user may purchase a certain business. The group of users ranked in the top 5%, 10%, and 30% by probabilities are treated as target groups.

Currently, Jiangsu Telecom employs a method that integrates multiple machine learning methods(e.g., xgboost, catboost, lightgbm) to build model. This study conducted experiments using autoregressive (LSTM) and non-autoregressive modules (iTransformer^26^ and TSMixer) based on CNGAN. The training data was collected from January 2023 to December 2023, and the test data was collected from January 2024 to February 2024. For the non-autoregressive method, time dimension embedding was used to model time series information. For all methods, city dimension embedding was used to model regional information. Before forward to the generator, the input was self-masked by noise to meet business requirements and the model was trained in parallel and step-wise.

The results are reported in Table 5. CosW indicates whether cosine dynamic weights are used in joint loss. The best results are indicated in black, and the second best are underlined. I indicates the percentage improvement of the best result relative to the integrated model.

**Table 5:** Results on Telecom market dataset.

Method	TOP%	Precision	Recall	F1
iTransformer w.o. CosW	5	0.136	0.113	0.123
	10	0.117	0.195	0.146
	30	0.099	0.493	0.164
TSMixer w.o. CosW	5	0.233	0.194	0.211
	10	0.178	0.297	0.223
	30	0.117	0.584	0.195
LSTM w.o. CosW	5	0.053	0.044	0.049
	10	0.056	0.094	0.070
	30	0.062	0.307	0.102
iTransformer w. CosW	5	0.389	0.324	0.353
	10	0.286	0.476	0.357
	30	0.147	0.732	0.244
TSMixer w. CosW	5	**0.597**	**0.497**	**0.542**
	10	**0.378**	**0.630**	**0.473**
	30	**0.157**	**0.782**	**0.261**
LSTM w. CosW	5	0.056	0.047	0.051
	10	0.067	0.112	0.084
	30	0.062	0.307	0.102
Current model	5	0.043	0.220	0.070
	10	0.034	0.350	0.061
	30	0.021	0.636	0.041
Improvement	5	55.41%	27.63%	47.27%
	10	34.38%	27.99%	41.12%
	30	13.53%	14.62%	22.01%

Bold and underlined text indicate the best and second-best performance, respectively.

From the results, it is evident that current model is weak. CNGAN using LSTM modules performs the worst, while CNGAN with non-autoregressive modules has achieved significant improvements. Specifically, CNGAN with TSMixer as the generator has comprehensively outperformed the integration model. The three indicators increased by 55.41%, 27.63%, and 47.27% for the top 5% user group, by 34.38%, 27.99%, and 41.12% for the top 10% user group, and by 13.53%, 14.62%, and 22.01% for the top 30% user group, as a result of average 31.5% increase. And the average improvement is 11.2% compared with CNGAN using iTransformer.

The deep learning method based on generative adversarial networks has comprehensively surpassed the current model. However, a major challenge of deep learning methods in business applications is their unexplained. The black box nature of deep learning fails to provide logical explanations, making it difficult to effectively integrate technology with experts experience. Although the current model has weaker forecasting ability, it offers relatively good explainability. Using tools such as SHAP^27^ (SHapley Additive exPlanations), it can provide explanations for the output probability, effectively helping business staff understand the modeling principles and the meaning of the output probabilities. This understanding aids in organizing marketing language and strategies. The method proposed in this paper can complement the current model, further assisting marketing units in improving marketing success rates. This has significant practical implications, as it combines the forecasting capability of deep learning with the explainability needed for practical business applications, thereby enhancing the overall effectiveness of marketing strategies.

## Other experiments and analysis

### Forecasting visualization

**Fig. 7:**
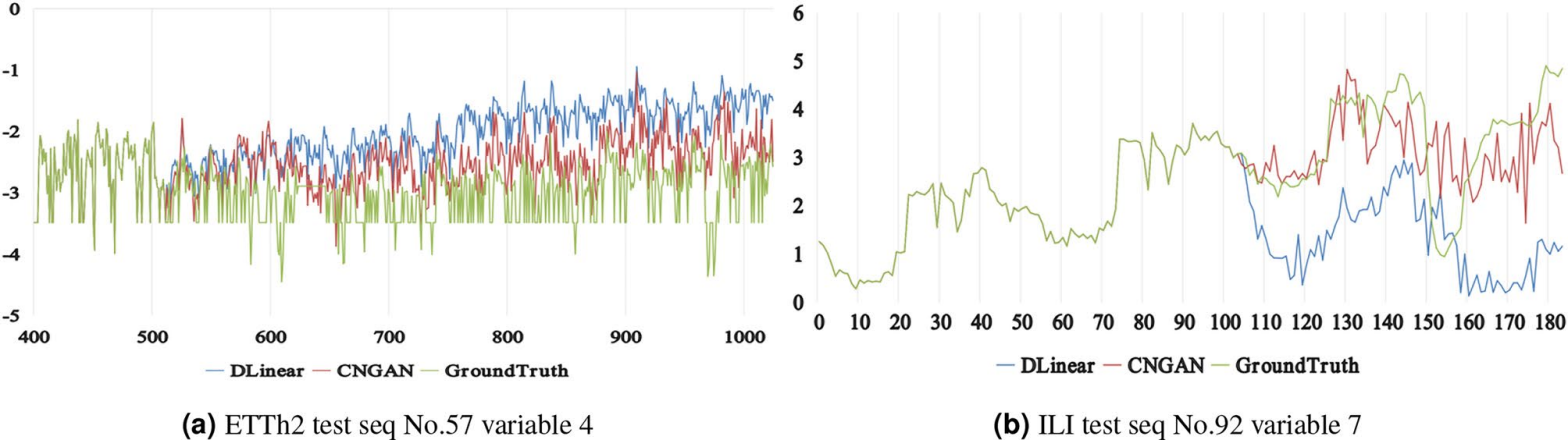
Results visualization. CNGAN achieves better fitting performance in time series forecasting compared to the baseline methods.

**Fig. 8:**
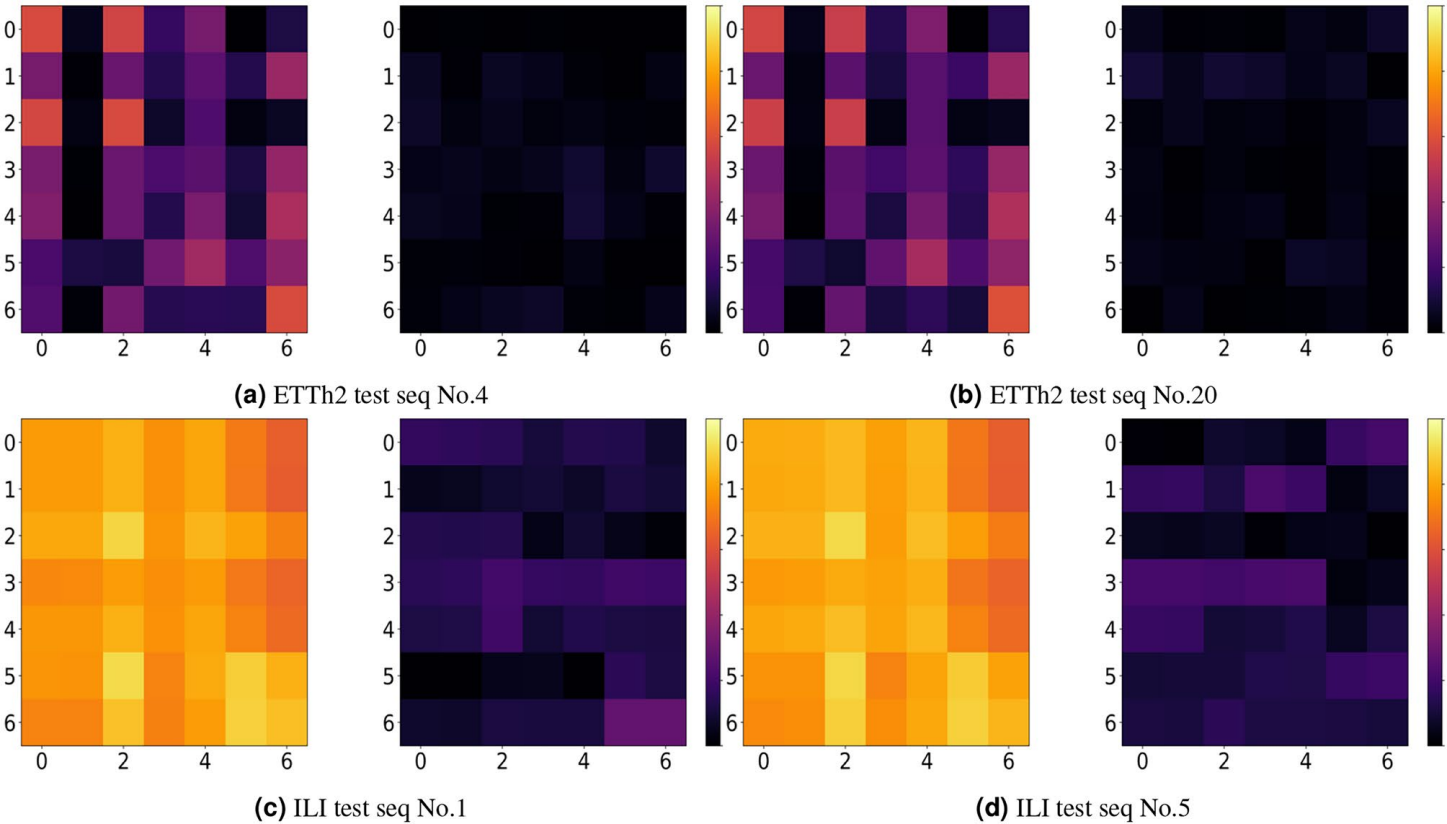
Noise visualization. For each group, the left shows the conditional noise, while the right shows the random noise. The conditional noise exhibits stronger correlation.

Figure 7 visualizes the results on the ETTh2 and ILI test split, comparing ground truth, CNGAN, and the baseline method DLinear. The visualization uses the 57th sequence and 4th variable with 512 history steps and 720 forecast steps in the test split on ETTh2. On ILI, it uses the 92th sequence and 7th variable with 104 history steps and 80 forecast steps in the test split. Due to length constraints, Fig. 8a truncates the visualization results.

The visualization results in Fig. 7 provide clear evidence of the superior performance of CNGAN over DLinear in time series forecasting. As shown in Fig. 8(a), although DLinear captures the overall temporal trend of the ETTh2 dataset, it exhibits significant deviations from the ground truth. CNGAN shows a markedly improved performance on the ETTh2 dataset. As shown in Fig. 8(b), DLinear do not able to capture the overall temporal trend, and it exhibits more significant deviations. However, CNGAN can not only capture the temporal trend, but also has a smaller deviation. The visualized results demonstrate the clear advantages of CNGAN over DLinear in long-term time series forecasting.

### Noise visualization

In this section, this study visualizes the impact of conditional noise and random noise for forecasting. Since this paper uses a linear generator, we consider the difference in linear correlation between pre-trained conditional noise and random noise with ground truth. The calculation process is as Eq. (14):

14 $$\begin{aligned} CM(i,j)=r(Z_i,Y_j,L) \end{aligned}$$

where $r(Z_i,Y_j,L)$ can be expressed as Eq. (15):

15 $$\begin{aligned} r(Z_i,Y_j,L)=|\frac{\sum _{k=0}^{L-1}(Z_{i,k}-\overline{Z_{i}})(Y_{j,k}-\overline{Y_{j}})}{\sqrt{\sum _{k=0}^{L-1}(Z_{i,k}-\overline{Z_{i}})^2}\sqrt{\sum _{k=0}^{L-1}(Y_{j,k}-\overline{Y_{j}})^2}}| \end{aligned}$$

where

16 $$\begin{aligned}&Z_i=Z(:,i) \end{aligned}$$

17 $$\begin{aligned}&Y_j=Y(:,j) \end{aligned}$$

Here, $CM$ represents the linear correlation matrix, *L* is sequence length, $Z_i$ and $Y_j$ are $i$-th column vector of noise matrix and $j$-th column vector of ground truth matrix respectively. This calculation measures the linear correlation between the $i$-th variable of the noise and the $j$-th variable of ground truth.

As shown in Fig. 8, we presents visualizations of the linear correlation matrix between conditional noise, random noise, and ground truth from the ETTh2 and ILI datasets. For ETTh2, sequences 4 and 20 from the test split are used, while for ILI, sequences 1 and 5 from the test split are used. The horizontal and vertical axes denote variable dimensions of two matrixs. Each position in the correlation matrix indicates the degree of linear correlation between corresponding multi-steps variables. The values closer to 0 indicate less correlation, while the values closer to 1 indicate higher correlation.

Figure 8a, b illustrates that random noise exhibits almost no linear correlation with the ground truth on the ETTh2 dataset. Conditional noise shows weak linear correlation with variable 1, but relatively high correlation in other variables. Figure 8c, d illustrates a large difference with the ILI dataset. Here, random noise maintains extremely low linear correlation across nearly all variable dimensions, whereas pre-trained conditional noise exhibits extremely high linear correlation with ground truth. These results intuitively demonstrate that a linear generator can easily generate more high-quality synthetic data from conditional noise, while generating from random noise is more challenging. Conditional noise provides crucial posterior information for the linear generator, enabling it to produce more accurate forecasting results.

### Ablation study

This paper quantitatively analyzes the impact of the proposed conditional noise and adversarial loss through ablation experiments. We remove the conditional noise and adversarial loss one by one and observe their effects on performance metrics. The results of the ablation experiments are summarized in Table 6, using the ETTh1, ETTh2, and ILI datasets, with the same evaluation metrics as the main experiment. For the ETT datasets, the forecasting steps are 720 and 1024, and for the ILI dataset, the forecasting steps are 60 and 80. The IMP. column records changes in metrics.

**Table 6:** Ablation results.

Datasets	Steps	Full		-Conditional noise			-Adversarial loss		
		MSE	MAE	MSE	MAE	IMP.	MSE	MAE	IMP.
ETTh1	720	0.444	0.461	0.462	0.483	− 0.040	0.467	0.485	− 0.047
	1024	0.519	0.507	0.518	0.513	− 0.005	0.525	0.518	− 0.017
ETTh2	720	0.477	0.483	0.668	0.574	− 0.282	0.766	0.620	− 0.426
	1024	0.642	0.555	0.829	0.642	− 0.274	0.862	0.663	− 0.328
ILI	60	1.744	0.894	2.153	1.032	− 0.547	2.423	1.102	− 0.887
	80	1.822	0.924	2.239	1.046	− 0.539	2.687	1.168	− 1.109

The ablation results show that removing either conditional noise or adversarial loss leads to a decrease in metrics. Overall, adversarial loss has a greater impact compared to conditional noise. From a data perspective, the influence of conditional noise and adversarial loss on the ETTh1 dataset is extremely small. However, on the ETTh2 and ILI datasets, both conditional noise and adversarial loss have a significant impact.

This work further investigates the impact of two key hyperparameters: the adversarial strength coefficient (alpha) and the kernel size of the 1D convolution used for temporal data. Considering computational efficiency, experiments were conducted on the relatively small ETTh2 dataset. Specifically, a grid search was performed over 11 values of alpha (ranging from 0.0 to 1.0 in increments of 0.1) and 11 values of kernel size (3, 10, 20, ..., 100), running each combination 10 times and averaging the performance metrics. Early stopping was used with a patience of 30, and each run took approximately $2\tilde{3}$ minutes, resulting in a total runtime of about 3 × 11 × 11 × 10 = 3630 minutes.

**Fig. 9:**
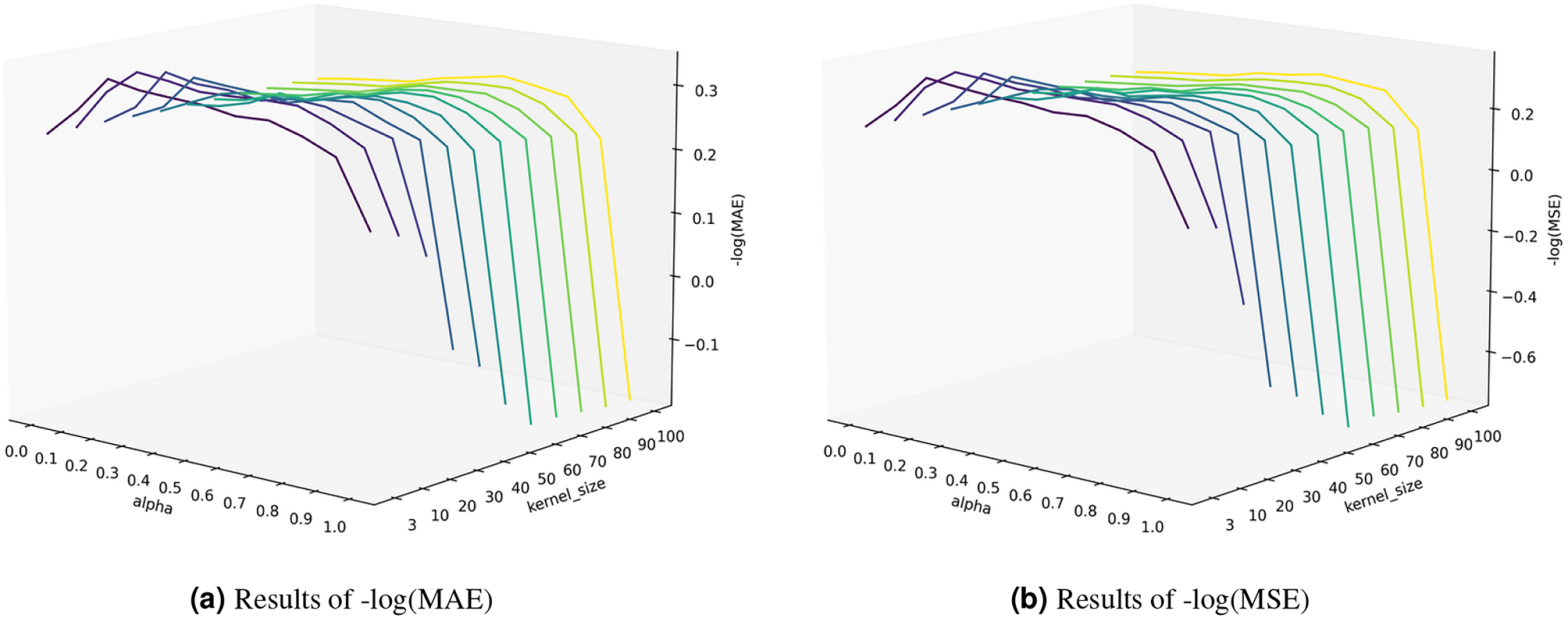
Visualization of the effects of key hyperparameters alpha and kernel size on model performance.

The results are illustrated in Fig. 9. For better readability, the MAE and MSE metrics were transformed into –log(MAE) and –log(MSE). The results were visualized in a three-dimensional space, where the x-axis represents alpha, the y-axis represents kernel size, and the z-axis corresponds to the transformed metric. Higher values on the z-axis indicate better performance. The experimental results demonstrate the influence of both hyperparameters, with consistent trends observed across both -log(MAE) and -log(MSE) metrics.

For alpha, the results generally show a trend which performance first improves and then declines as adversarial strength alpha increases. The optimal alpha is found to be between 0.2 and 0.3. At the two extreme points, when alpha = 0.0 (purely supervised training), the best performance is not achieved, and when alpha = 1.0 (pure adversarial training), the performance significantly drops. These results highlight the importance of Supervised signals in the CNGAN framework. Overall, these findings underscore the effectiveness of combining adversarial training with supervised learning in a hybrid training approach.

Regarding the 1D convolution kernel size, larger kernels generally lead to performance degradation, suggesting that fine-grained convolutions are more beneficial for processing temporal data. It is also observed that the sensitivity to kernel size varies with the value of alpha: the model is more sensitive to kernel size when the adversarial strength is lower and less sensitive when the adversarial strength is higher. This indirectly demonstrates the robustness and scalability of the generative adversarial network architecture for time-series data. One potential direction for future work is to further exploit this property to optimize model performance.

### What traffic happened?

A more in-depth investigation was conducted to analyze the low performance on the traffic dataset. The traffic dataset is substantially larger than the other datasets used in this study. Specifically, it contains approximately 1.79 times more data than the electricity dataset and 2236 times more than the ILI dataset. Such high information density may exceed the discriminator’s capacity, resulting in weaker feedback during adversarial training. To facilitate further experimentation, an A100 GPU was utilized. Earlier experiments were limited by the memory constraints of a 3070Ti GPU, which restricted the use of large batch sizes, thereby reducing training efficiency and scalability. With the enhanced computational resources, a comparative analysis of training dynamics was performed between the traffic and ETTh2. The normalized gradients provided from both the generator and the discriminator were monitored throughout the training process.

**Fig. 10:**
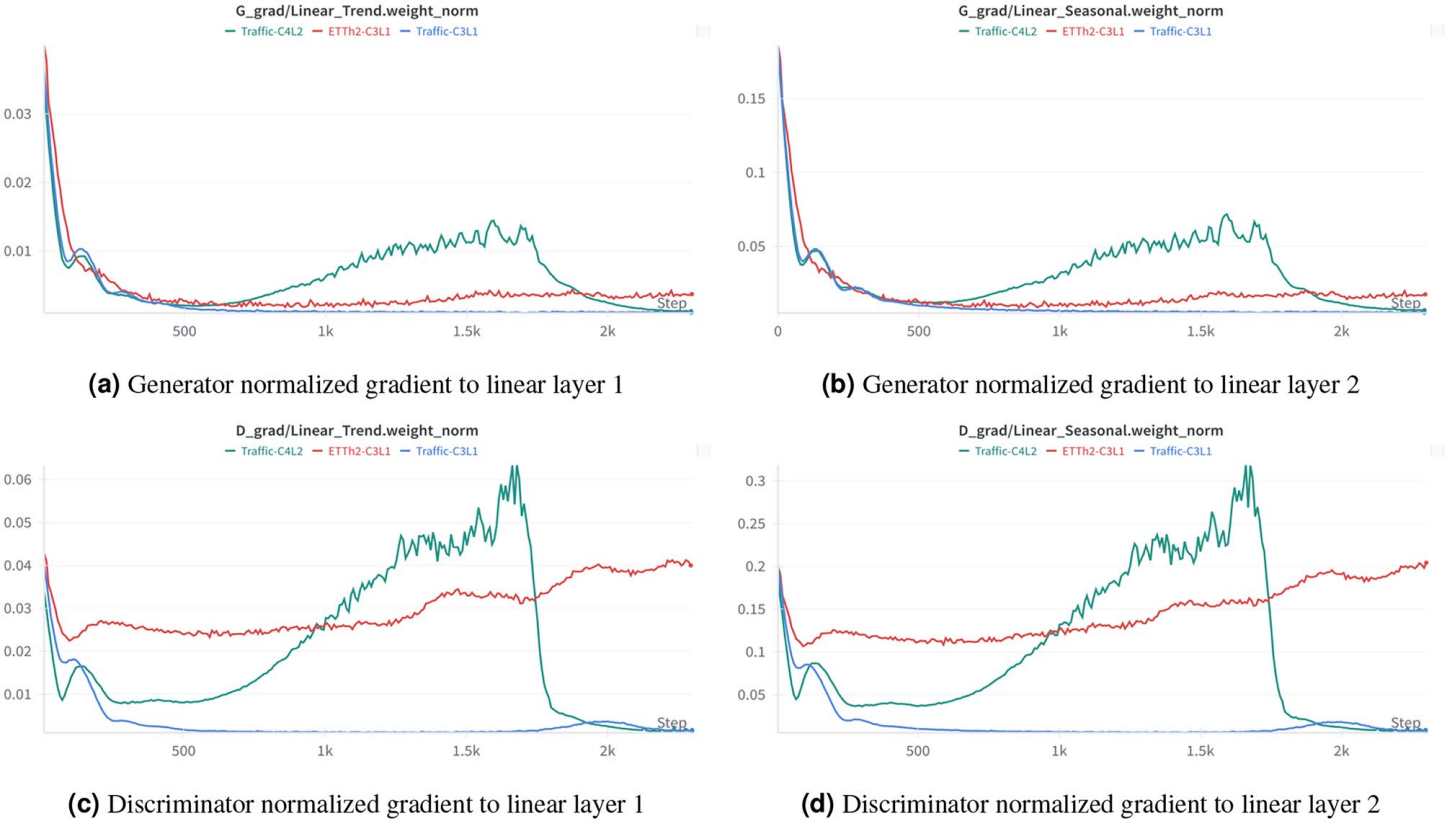
Normalized gradients during training process.

Figure 10 shows the normalized gradients provided from both the generator and the discriminator during the training process. The normalized gradient magnitude serves as an indicator of the strength of the learning signal each component contributes during optimization. C3L1 means that a discriminator comprises three convolutional layers and one fully connected layer, while C4L2 consists of four convolutional layers and two fully connected layers.

First, it was observed that during GAN training, the discriminator contributes the majority of the gradient signal, relative to the generator, emphasizing its critical role in driving effective model optimization. Furthermore, the results indicate that dataset complexity has a significant impact on the gradient signal strength from the discriminator. The same generator exhibits significantly different performance on ETTh2 and traffic datasets. This suggests that, on complex datasets such as traffic, lightweight discriminator is more susceptible to gradient vanishing. As shown in Table 7, at 720 forecasting steps, increasing the depth of the discriminator alleviate this issue to some extent.

**Table 7:** MAE and MSE metrics of different discriminator architectures at 720 forecasting steps.

Discriminator Architecture	MAE	MSE
C3L1	0.706	1.169
C4L1	0.604	0.743
C5L1	0.611	0.758
C3L2	0.554	0.605
C3L3	0.496	0.508
C4L1	0.421	0.487
C4L2	**0.334**	**0.430**
C4L3	0.356	0.498

Bold and underlined text indicate the best and second-best performance, respectively.

Compared to the C3L1 discriminator architecture, scaling the discriminator resulted in varying degrees of improvement. In the experiments conducted in this study, the best results were achieved with a discriminator consisting of four convolutional layers and two fully connected layers. This observation suggests that exploring how to adjust or replace the discriminator with more effective architectures is a promising direction for future research.

## Conclusion

This study proposes CNGAN to prove the feasibility of generative methods in long-term time series forecasting. CNGAN introduces conditional noise and SNN discriminator. Visualization results demonstrate that conditional noise effectively encodes posterior information, enhancing the generator ability to capture context. The SNN discriminator naturally models the adversarial aspect of generative adversarial networks. It is confirmed that combining supervised learning with unsupervised adversarial learning can improve model performance by ablation experiments. Visualization of forecasting results shows that CNGAN outperforms the DLinear method while maintaining simple characteristic. Furthermore, this study explores the practical implications in Telecom market scenarios. Research results verify that the forecasting capability of CNGAN can enhance the accuracy of marketing recommendations. Future research could explore the combination of other encoder and decoder modules to further extend the application of generative adversarial networks into new fields. To the best of our knowledge, this work is one of the first studies to utilize the generative adversarial framework for long-term time series forecasting. We hope this study will inspire further research, open up new directions and methodologies, and provide valuable references for the academic community. Moreover, it also offers practical solutions that can be applied to real-world scenarios.

## Data Availability

The datasets generated and/or analysed during the current study are not publicly available due to privacy but are available from the corresponding author on reasonable request.
